# Expression of SHH signaling molecules in the developing human primary dentition

**DOI:** 10.1186/1471-213X-13-11

**Published:** 2013-04-08

**Authors:** Xuefeng Hu, Shuo Zhang, Guimiao Chen, Chensheng Lin, Zhen Huang, YiPing Chen, Yanding Zhang

**Affiliations:** 1 Fujian Key Laboratory of Developmental and Neuro Biology, College of Life Science, Fujian Normal University, Fuzhou, Fujian, 350108, P.R. China; 2 Department of Cell and Molecular Biology, Tulane University, New Orleans, LA, 70118, USA

**Keywords:** Deciduous tooth, Tooth development, Gene expression, SHH, PTC, SMO, GLI

## Abstract

**Background:**

Our current knowledge on tooth development derives primarily from studies in mice. Very little is known about gene expression and function during human odontogenesis. Sonic Hedgehog (SHH) signaling has been demonstrated to play crucial roles in the development of multiple organs in mice, including the tooth. However, if SHH signaling molecules are expressed and function in the developing human embryonic tooth remain unknown.

**Results:**

We conducted microarray assay to reveal the expression profile of SHH signaling pathway molecules. We then used in situ hybridization to validate and reveal spatial and temporal expression patterns of a number of selected molecules, including *SHH*, *PTC1*, *SMO*, *GLI1, GLI2,* and *GLI3,* in the developing human embryonic tooth germs, and compared them with that in mice. We found that all these genes exhibit similar but slightly distinct expression patterns in the human and mouse tooth germ at the cap and bell stages.

**Conclusions:**

Our results demonstrate the operation of active SHH signaling in the developing human tooth and suggest a conserved function of SHH signaling pathway during human odontogenesis.

## Background

Tooth development in mice and humans shares striking morphological similarities, undergoing through the lamina, bud, cap, and bell stages [1]. In the developing human embryo, the thickened dental epithelia first appear on the upper and lower jaws at the sixth week of gestation. Around the seventh week of gestation, these thickenings undergo localized cell proliferation to form the epithelial bud in the subjacent mesenchyme, and reach the cap stage at the tenth week. At the fourteenth week of gestation, tooth development progresses to the bell stage and begins cytodifferentiation [1,2]. In the past decade, rapid progress has been made towards understanding of molecular mechanisms underlying tooth development. However, the studies were conducted mainly in mice. Despite the morphological similarities of tooth development in mice and humans, little is known about whether or not similar molecular mechanisms are employed during human odontogenesis [3].

Sonic Hedgehog (SHH), a mammalian homologue of Drosophila secreted morphogen Hedgehog (Hh), plays many key roles in the development of multiple organs [4]. SHH signals through its transmembrane receptors Patched (PTC) and Smoothened (SMO) [5,6]. In the absent of SHH, PTC inhibits the SMO activity for signaling transduction. Binding of SHH protein to PTC releases this inhibition, allowing SMO to activate the downstream signaling cascade. The zinc finger transcription factors GLI1-3 mediate the SHH signaling to regulate the expression of downstream target genes [7,8]. More recently, additional components in the Hedgehog signaling pathway have been identified, including Hedgehog-interacting protein (Hhip, also known as Hip1), a negative regulator of Hedgehog signaling [9], and the glycoprotein Gas1 (growth arrest-specific gene), an antagonistic effector of Hedgehog signaling [10].

In mice, Shh, its receptors, and downstream effectors are expressed and play pivotal roles in every stage of tooth development, including tooth initiation, patterning, and growth [11-18]. Targeted inactivation of *Shh* leads to retarded grown and mis-patterned teeth in mice [13], indicating an essential role for Shh in tooth morphogenesis.

The rapid expansion of our knowledge makes tooth regeneration in the human a realistic possibility in the near future. Profiling gene expression in normal human tooth development is a prerequisite for studying tooth regeneration or reconstitution of a bioengineered tooth organ. In the present study, we examined the expression profiles and patterns of SHH signaling molecules in the developing human embryonic tooth germ and compared them with that in mice.

## Results

### Gene expression profiles of SHH signaling molecules in the cap stage human primary dentition

To profile gene expression in the developing human tooth germ, we conducted cDNA microarray assays on human embryonic tooth germ at the cap stage. We selected genes that are known to be involved in SHH signaling pathway, including *SHH*, *PTC1*, *PTC2*, *SMO*, *GLI1-3*, *GAS1*, *HHIP*, *SUFU*, and *STK36*, and compared their expression levels with that in the lip tissue. The lip tissue was selected as control, because it is a non-dental tissue closer to the tooth germ. Fold changes of gene expression are presented in Table 1. Among them, *SHH* had the highest level of expression in all three types of primary dentition (incisor, canine, and the first premolar) examined, while GAS1 exhibited a lowest level of expression in the tooth germs, as compared with that in the lip tissue. The rest genes also exhibited various increased levels of expression, from 1 to 4 folds, as compared with the expression level of each corresponding gene in the lip tissue. These results demonstrated a relatively high expression level of SHH signaling molecules in the developing human primary dentition, and prompted us to validate and examine the expression patterns of several key molecules and compare them with that in the developing mouse tooth.

**Table 1:** Microarray expression profiling of SHH signaling in human tooth germ at the cap stage

**Name**	**Description**	**Gene bank**	**Fold change (P-value)**		
			**Molar**	**Incisor**	**Canine**
SHH	sonic hedgehog	AI192528	7.291(0.011) *	8.610(0.044) *	4.671(0.032) *
PTCH1	patched 1	BG054916	3.356(0.003) **	3.380(0.002) **	4.249(0.002) **
PTCH2	patched 2	NM_003738	0.805(0.975)	1.522(0.422)	1.015(0.368)
SMO	Smoothened	NM_005631	1.052(0.010) **	1.586(0.317)	1.156(0.160)
GLI1	GLI family zinc finger 1	NM_005269	1.537(0.009) **	1.701(0.028) *	2.426(0.030) *
GLI2	GLI family zinc finger 2	NM_030379	1.135(0.042) *	1.258(0.193)	1.262(0.098)
GLI3	GLI family zinc finger 3	AW021102	1.854(0.001) **	1.580(0.083)	1.607(0.039) *
GAS1	growth arrest-specific 1	AI161237	0.635(0.007) **	0.640(0.784)	0.507(0.480)
HHIP	hedgehog interacting protein	AK098525	3.852(0.021) *	4.297(0.019) *	2.666(0.011) *
SUFU	suppressor of fused	AF144231	1.315(0.224)	2.628(0.131)	2.027(0.051)
STK36	serine/threonine kinase 36	AU149216	1.858(0.001) **	1.762(0.073)	1.531(0.039) *

*: (P value ≤0.05, *t*-test); **: (P value ≤0.01, *t*-test).

### Expression of *SHH* in the developing human primary tooth germ

**Figure 1:**
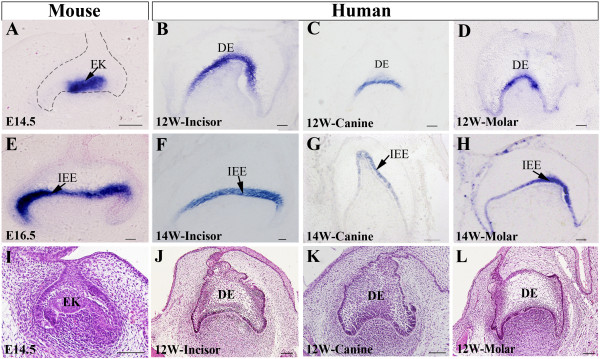
**Expression of *SHH *in human and mouse tooth germ at the cap and bell stages.** (**A-D**) *SHH* expression at the cap stage. (**A**) E14.5 mouse molar; (**B**) 12-week human primary incisor; (**C**) 12-week human primary canine; (**D**) 12-week human first premolar molar. (E-H) *SHH* expression at the bell stage. (**E**) E16.5 mouse molar; (**F**) 14-week human primary incisor; (**G**) 14-week human primary canine; (**H**) 14-week human primary first premolar molar. (**I-L**) Histology of mouse and human tooth germs at the cap stage. (**I**) E14.5 mouse molar; (**J**) 12-week human primary incisor; (**K**) 12-week human primary canine; (**K**) 12-week human primary first premolar. DE, dental epithelium; EK, enamel knot; IEE, inner enamel epithelium. Scale bar = 100 μm.

In mice, *Shh* has been demonstrated to be expressed in a restricted pattern in the developing tooth germ [11,12,19,20]. At the E11.5 laminar stage, *Shh* begins to be expressed in the epithelium of the dental placodes. At the E14.5 cap stage, *Shh* is exclusively expressed in the enamel knot, the signaling center of developing tooth (Figure 1A). By the E16.5 bell stage, *Shh* expression is restrictedly present in the pre-ameloblasts (inner enamel epithelium) (Figure 1E). In the developing human tooth germs at the cap sage, *SHH* expression exhibited a restricted expression pattern in the dental epithelium of incisor, canine, and the first premolar molar (Figure 1B-D). Interestingly, in the developing human primary dentition at this stage, there does not exist a typical enamel knot morphologically resembling that seen in the mouse (Figure 1I-L). *SHH* transcripts were most notably present in the region corresponding to the enamel knot. At the bell stage, *SHH* expression was observed in the inner enamel epithelium in the human primary dentition (Figure 1F-H). The similar expression pattern of *SHH* in the developing human primary dentition as compared with that in mice suggests a conserved role for SHH signaling during human odontogenesis.

### Expression of *PTC1*and *SMO* in the developing human primary dentition

**Figure 2:**
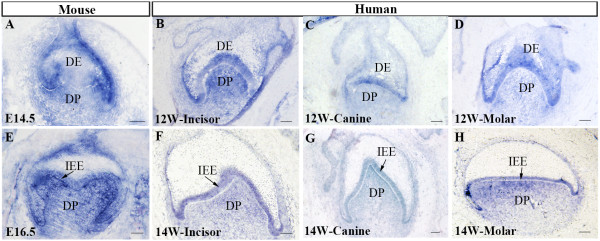
**Expression patterns of *PTC1 *in the developing human and mouse tooth germs at the cap and bell stages.** (**A**) *Ptc1* expression in E14.5 mouse molar. (**B**) *PTC1* expression in 12-week human primary incisor. (**C**) *PTC1* expression in 12-week human primary canine. (**D**) *PTC1* expression in 12-week human first premolar molar. (**E**) *Ptc1* expression in E16.5 mouse molar. (**F**) *PTC1* expression in 14-week human primary incisor. (**G**) *PTC1* expression in 14-week human primary canine. (**H**) *PTC1* expression in human primary first premolar molar. DE, dental epithelium; DP, dental papilla; IEE, inner enamel epithelium. Scale bar = 100 μm.

**Figure 3:**
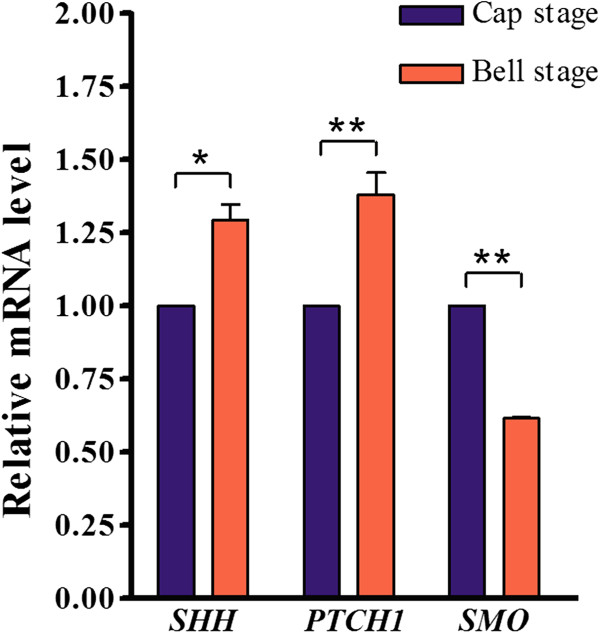
**Changes in *SHH***, ***PTC1*, and *SMO *expression level in developing human primary dentition at the cap and bell stages.** Real-time RT-PCR results show changes in expression levels of *SHH*, *PTC1*, and *SMO* at the cap and bell stages of the developing human primary dentition. The expression levels of each gene at the cap stage were set at one. *: P value ≤0.05; **: P value ≤0.01

As a Shh receptor and downstream target, *Ptc1* expression is always associated with *Shh* in developing organs. At the cap and bell stages of tooth development in the mouse, *Ptc1* has a widespread expression pattern in both the dental epithelium and dental mesenchyme (Figure 2A, 2E) [21], suggesting the long range and widespread effect of Shh signaling in the developing tooth. In the developing human primary dentition, the expression pattern of *PTC1* basically resembled that in the mouse at the cap stage (Figures 2B-D) and the bell stage (Figure 2F-H), indicating an active SHH signaling in the developing human embryonic tooth germs. However, in the mouse, *Ptc1* expression is equally strong in the inner and outer enamel epithelium, but in the human primary dentition, the outer enamel epithelium of the developing human tooth germ presented a relatively lower level of *PTC1* expression, as compared to *PTC1* expression level in the inner enamel epithelium. These observations indicate similar but slightly distinct *PTC1* expression pattern in the developing human and mouse tooth germs. We also examined the expression patterns of *SMO* in the human developing dentition. Similar to the expression pattern of *PTC1*, *SMO* transcripts were also detected in the dental epithelium and the dental papilla of all three types of the primary tooth germs at the cap and bell stages (Figure 3). This expression pattern of *SMO* is identical to that of its counterpart in the developing mouse tooth germ [12].

**Figure 4:**
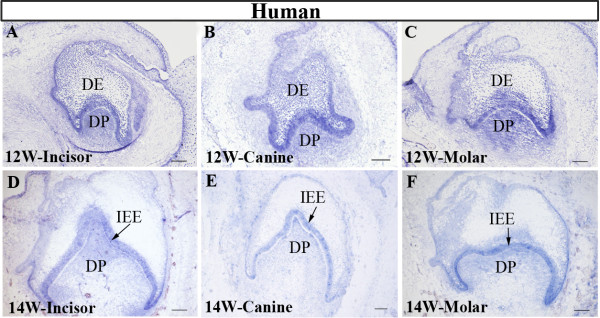
**Expression patterns of *SMO *in the developing human and mouse tooth germs at the cap and bell stages.** (**A**) *SMO* expression in 12-week human primary incisor. (**B**) *SMO* expression in 12-week human primary canine. (**C**) *SMO* expression in 12-week human first premolar molar. (**D**) *SMO* expression in 14-week human primary incisor. (**E**) *SMO* expression in 14-week human primary canine. (**F**) *SMO* expression in human primary first premolar molar. DE, dental epithelium; DP, dental papilla; IEE, inner enamel epithelium. Scale bar = 100 μm.

To further confirm the expression levels of *SHH*, *PTC1*, and *SMO* in the developing human primary dentition at these two stages, we conducted real-time RT-PCR to determine the expression levels of these genes. We pooled incisor, canine, and the first premolar germs from each embryonic mandibular quadrant and isolated total RNAs from each pool for real-time RT-PCR. As shown in Figure 4, the changes in expression levels of these genes at the cap and bell stages basically matched with that observed by in situ hybridization.

### Expression of *GLI1*, *GLI2*, and *GLI3* in the developing human tooth germ

**Figure 5:**
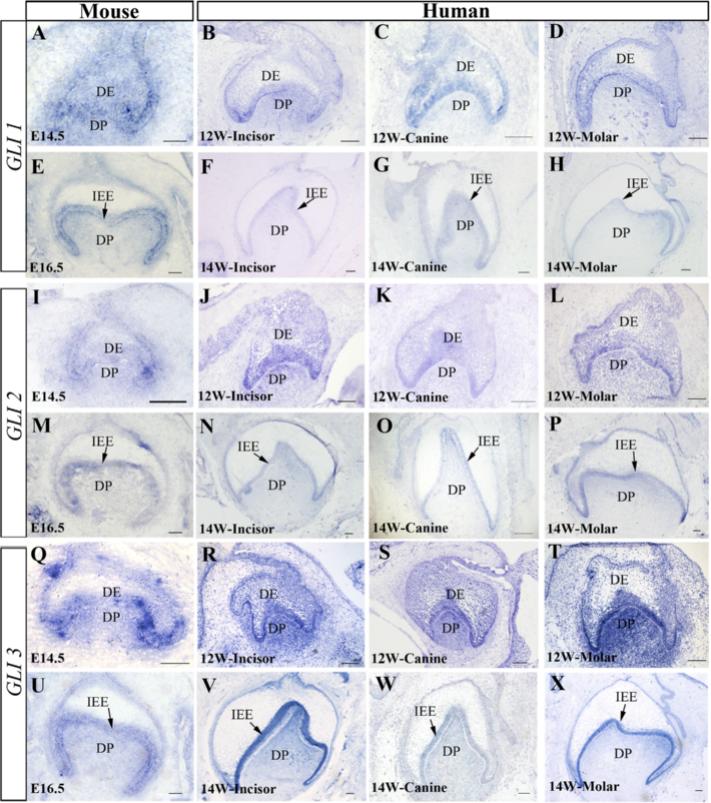
**Expression patterns of *GLI1, GLI2 *and *GLI3 *in the developing human and mouse tooth germs at the cap and bell stages.** (**A-H**) *GLI1* expression in the mouse (**A, E**) and human (**B-D**, **F-H**) tooth germs at the cap (**A-D**) and bell (**E-H**) stages. (**I-P**) *GLI2* expression in the mouse (**I, M**) and human (**J-L, N-P**) at the cap (**I-L**) and bell (**M-P**) stages. (**Q-X**) *GLI3* expression in the mouse (**Q, U**) and human (**R-T, V-X**) at the cap (**Q-T**) and bell (**U-X**) stages. DE, dental epithelium; DP, dental papilla; IEE, inner enamel epithelium. Scale bar = 100 μm.

The GLI transcription factors function as downstream effectors of SHH signaling. We extended our studies to examine and compare the expression of *GLI1*, *GLI2*, and *GLI3* in the developing tooth in mice and humans. At the cap stage, *GLI1* expression was detected in the dental epithelium and mesenchyme in a similar pattern in both human and mouse tooth germ with relatively lower level in the dental papilla in the human (Figure 5A-D). At the bell stage, *Gli1* expression remained in the inner enamel epithelium and the dental papilla of the mouse molar (Figure 5E). However, *GLI1* expression became much weaker in the inner enamel epithelium of the human primary dentition, with above background signals in the dental papilla, as compared with its expression at the cap stage (Figure 5F-H). *GLI2* also exhibited a similar expression pattern in the human tooth germ as compared to that in mice. At the cap stage, *GLI2* was found to be expressed in the dental epithelium and mesenchyme (Figure 5I-L). At the bell stage, *GLI2* expression was detected primarily in the inner enamel epithelium, with a lower level in the dental mesenchyme (Figure 5M-P). An overall similar expression pattern of *GLI3* in the developing tooth germs was found in both mice and humans. In mice, a relatively strong *Gli3* expression was found in the dental epithelium and mesenchyme at the cap stage, but the expression level became reduced in the mesenchyme at the bell stage (Figure 5Q, 5U). Similarly, in the developing human dentition, strong *GLI3* expression was observed in the inner enamel epithelium and mesenchyme at the cap stage (Figure 5R-T), but at the bell stage, the expression became relatively weaker (Figure 5V-X). These observations indicate similar expression patterns of *GLI* genes in the developing human and mouse tooth germs.

## Discussion

Development of the mouse tooth serves as an excellent model to study the molecular mechanism of mammalian organogenesis. The mouse tooth develops through sequential and reciprocal interactions between the dental epithelium and dental mesenchyme. Signaling molecules are known to mediate such epithelial-mesenchymal interactions [22-24]. In the mouse, Shh signaling pathway has been shown previously to exert pivotal roles at various steps during tooth development. Prior to epithelial invagination, the expression of *Shh* in the dental epithelium suggests a role of this pathway for tooth germ initiation. Repression of *Shh* expression at the tooth forming site in the oral ectoderm by ectopic expression of *Wnt-7b* inhibited tooth development [14]. At the tooth initiation stage, *Shh* may acts as a mitogen to induce cell proliferation, thus regulating the ingrowth of the epithelial tooth bud [25]. At the cap stage, *Shh* expression is restricted in the enamel knot, the signaling center of tooth development, implicating a role for *Shh* in the patterning of tooth cusps. During the bell stage, *Shh* expression spreads to the surrounding inner enamel epithelium and is maintained in differentiating ameloblasts. Conditional inactivation of *Shh* shortly after ingrowth of dental epithelium led to reduction of the tooth germ size with severely disrupted morphology. However, both odontoblastic and ameloblastic specific markers were expressed and extracellular matrix was produced [13]. These results suggest that *Shh* may not be necessary for terminal cytodifferentiation of odontoblasts or ameloblasts. *Ptc, Smo* and *Gli* are the major components in the Shh signaling pathway. The fact that they are all expressed in the dental epithelium and the dental mesenchyme suggests that Shh signaling is involved in not only intra-epithelial interaction but also epithelial-mesenchymal interaction during tooth development [11,12]. In humans, mutations in SHH leads to solitary maedian maxilarry central incisor in addition to holoprosencephaly [26]. Shh signaling is thus absolutely required for odontogenesis in the mouse and human.

In the current studies, using microarray analysis, we showed that many critical components involved in the SHH signaling pathway, including the ligand (SHH), receptors (PCT1, and PCT2), transducers (SMO, SUFU, and STK36), antagonists (Hip1, and Gas1), and transcription factors (GLI1, GLI2, and GLI3) were present at relatively higher levels in the human primary dentition, indicating the operation of active SHH signaling during human odontogenesis. Whereas, Shh pathway is considered not to be active or at least remain at very low level in the lip tissue. In situ hybridization examination further validated the expression of a number of selected genes, and revealed their spatial and temporal expression patterns in the developing human tooth germ at the cap and bell stages. The similar expression patterns of these molecules in both human and mouse developing teeth suggest a conserved function of SHH signaling pathway in human tooth development.

The development, patterning, and organization of dentition in mammals share many similarities. However, it is also characteristic for each species. The tooth is an ectodermal organ that develops through epithelial-mesenchymal interactions. Several families of signaling molecules including TGFβ, WNT, FGF, and Hedgehog are known to mediate these interactions and are used repeatedly during the process of tooth development. Despite high similarities in human and mouse odontogenesis [1], there are some noticeable differences. For example, developmental phase and tooth size are significantly different. In mice, the developmental phase from tooth initiation (E11.5) to tooth eruption (postnatal day 11) is about 20 days. In humans, however, this process takes about 400 days [2]. Our previous [3] and present studies demonstrate that many genes known to be critical for tooth development in mice exhibit similar expression patterns in both the human and mouse, suggesting similar, if not identical, mechanisms and networks that are employed in the regulation tooth development in humans. However, some genes that were examined also exhibited differential expression patterns, as exemplified by the persistent expression of FGF8 in the dental epithelium and mesenchyme throughout the entire tooth developmental process in the humans [3,27] and by the slightly different expression patterns of *PTC1* and *GLI* genes in this study. Thus our results support the idea that the diversity of tooth types and dental patterns may have resulted from tinkering with the conserved signal pathways during evolution [24]. Nevertheless, unveiling gene expression profiles in the developing human tooth will serve not only as a prerequisite for future investigation of gene function and genetically related dental diseases, but also as a solid foundation for tooth regeneration studies in humans.

## Conclusions

SHH signaling molecules are expressed in the developing human primary dentition at a higher level compared to the adjacent tissue. Several key molecules of SHH signaling including SHH, PTC1, SMO, GLI1-3 exhibit similar expression patterns in the developing human and mouse tooth germs. *GLI* and *PTC1* genes show slightly distinct expression in the developing human primary dentition as compared to that in mice. Our results demonstrate an active SHH signaling that operates in the developing human tooth and suggest a conserved function of SHH signaling pathway during human odontogenesis.

## Methods

### Samples

Human embryonic tissues of 10^th^ to 14^th^ week gestation from chemically induced termination of pregnancy were provided by the Hospital for Woman and Children Health of Fujian Province. Written informed consent was obtained from participants for the use of their medically aborted embryos for scientific research. The embryonic tissues were either subjected to tooth germ isolation for microarray assays or were fixed in 4% paraformaldehyde at 4°C overnight and then processed for paraffin embedding and sectioning at 10 μm. The use of human embryonic tissues in this study was ethically permitted by the Ethics Committee of Fujian Normal University.

### Microarray analysis

Tooth germs of molar, incisor, and canine at the cap stage were dissected, respectively, from 12-week-old human embryonic oral cavity. To screen for factors that are preferentially expressed in dental tissues, we included a piece of lip tissue dissected from the same embryo for comparison. Total RNA was isolated from these human orimary tooth germs and lip tissues with QIAGEN RNAeasy mini kit. RNA samples were isolated from the tooth germs of three different human embryos and analyzed for biological triplicates. Gene expression profiles were examined on Affymetrix U133 plus 2.0 array chip containing approximately 54000 genes (Affymetrix, Santa Clara, CA, USA). Microarray data was read with Affymetrix scanner 3000 and gene expression levels were analyzed with GCOS1.4. Components of SHH signaling pathway were abstracted manually according to literatures. The gene expression level, fold change and the statistical significance (P value) was calculated by R software environment (http://www.R-project.org). Data of fold changes were presented as compared with the expression levels of each corresponding gene in the lip tissue.

### In situ hybridization

Human cDNA clones of *PTC1* (777 bp), *SMO* (723 bp), *GLI1* (656 bp), *GLI2* (439 bp), and *GLI3* (522 bp) were purchased from Open Biosystems (Thermo Scientific Inc). A 1576-bp human *SHH* cDNA was a gift from Dr C. Tabin of Harvard Medical School. cDMA fragments were subcloned into *pBluesscript* for riboprobe transcription. Mouse *Shh*, *Ptch1*, *Patch2*, *Smo*, *Gli1*, *Gli2,* and *Gli3* probes were used to detect gene expression in the mouse tooth germ [12,28]. Section in situ hybridization with DIG-labeled riboprobe was carried out as described previously [3,28].

### Real-time PCR

Human embryonic tooth germs at the cap and bell stages were isolated and all three types of tooth germs (incisor, canine, and premolar) from one quadrant were pooled and subjected to total RNA extraction using RNeasy® Mini Kit (QIAGEN) according to the manufacturer’s instructions. Template cDNAs were synthesized by a standard reverse transcript reaction (RT) using oligo(dT) primer and RevertAid™ M-MuLV Reverse Transcriptase (Fermentas, EU). 1ug of total RNA was used for RT reactions. Real-time PCR was performed with DyNAmo ColorFlash SYBR Green qPCR Kit (Thermo Scientific), and signals were detected by ABI 7300 Real-time PCR System (Applied Biosystems). GAPDH mRNA was detected for each sample as internal control and was used to correct for target genes. The same primers were used in this experiment as reported previously [29]. Relative mRNA expression levels of target genes in human bell stage tooth germs were achieved using the △△CT method in comparison with the cap stage. Relative mRNA expression levels were shown as Mean and SD from three independent experiments. Student’s *t*-test was analysis though “Two-way ANOVA” using the GraphPad Prism5 software.

## Competing interests

The authors declare that they have no competing interests.

## Authors’ contributions

XH carried out the microarray, in situ studies and drafted the manuscript. ZS participated in in situ experiments. GC, CL and ZS participated in in situ experiments and making illustrations. ZH performed the statistical analysis. YPC and YZ conceived the study, participated in its design and coordination, and helped to draft the manuscript. All authors read and approved the final manuscript.
